# Occurrence of Contaminants in Groundwater from the Central–Northeastern Part of the Romanian Plain and the Associated Risk Assessment

**DOI:** 10.3390/toxics14070638

**Published:** 2026-07-21

**Authors:** Crinela Dumitrescu, Claudia Stihi, Roxana Elena Ionete, Elisabeta-Irina Geană, Corina Teodora Ciucure, Petre Brețcan

**Affiliations:** 1 Faculty of Sciences and Arts, Valahia University of Targoviste, 13 Aleea Sinaia St., 130004 Targoviste, Romania; 2 Academy of Romanian Scientists, 3 Ilfov St., 050044 Bucharest, Romania; 3 National Research and Development Institute for Cryogenic and Isotopic Technologies, 4 Uzinei St., 240050 Ramnicu-Valcea, Romania; roxana.ionete@icsi.ro (R.E.I.); irina.geana@icsi.ro (E.-I.G.); corina.ciucure@icsi.ro (C.T.C.); 4 Faculty of Humanities, Valahia University of Targoviste, 35 Stancu Ion St., 130105 Targoviste, Romania; petre.bretcan@valahia.ro

**Keywords:** polycyclic aromatic hydrocarbons, petrogenic/pyrogenic source, diagnostic ratio method, ecological risk, carcinogenic/non-carcinogenic risk

## Abstract

Groundwater is a vital resource for drinking water supply, agriculture, and industrial activities. However, contamination by organic pollutants may pose significant threats to both ecosystem integrity and human health. This study assessed the environmental and human health risks associated with polycyclic aromatic hydrocarbons (PAHs) in groundwater collected from 27 localities in the central–northeastern Romanian Plain. The concentrations of naphthalene, acenaphthene, fluorene, phenanthrene, anthracene, fluoranthene, pyrene, benzo[a]anthracene, chrysene, benzo[b]fluoranthene, benzo[k]fluoranthene, benzo[a]pyrene, dibenzo[a,h]anthracene, benzo[g,h,i]perylene, and indeno [1,2,3-cd]pyrene were determined using high-performance liquid chromatography coupled with fluorescence detection. Benzo[a]pyrene concentrations ranged from <LOD to 0.08 ng/L, while total concentrations varied between 2.56 to 11.2 ng/L. The environmental risk associated with groundwater contamination was classified as low to moderate, with total risk coefficients ranged from 0.2 to 2.3. Potential contamination sources were identified using the diagnostic ratio method, complemented by multivariate statistical analysis, indicating predominantly pyrogenic and mixed pyrogenic–petrogenic sources. Human health risk assessment indicated that groundwater ingestion posed neither non-carcinogenic risks (Hazard Index, HI < 1) nor unacceptable carcinogenic risks. The Incremental Lifetime Cancer Risk (ILCR) values ranged from 5.7 × 10^−9^ to 2.2 × 10^−8^ for infants, 2.7 × 10^−9^ to 1.0 × 10^−8^ for children, and 1.5 × 10^−9^ to 6.1 × 10^−9^ for adults.

## 1. Introduction

Polycyclic aromatic hydrocarbons (PAHs) are ubiquitous environmental contaminants composed of two or more fused aromatic rings and are recognized among the most important classes of persistent organic pollutants. They are generated primarily through the incomplete combustion of organic materials, including coal, petroleum, natural gas, wood, waste, biomass, tobacco, and charbroiled food, but are also emitted from vehicle emissions, cigarette smoke, and natural processes such as wildfires and volcanic eruptions [1,2,3,4,5,6].

PAHs are characterized by low aqueous solubility, pronounced lipophilicity, and remarkable chemical and thermal stability [2,3,4,5,6,7,8,9,10,11,12,13,14,15,16]. Some low-molecular-weight PAHs are semi-volatile and may occur in both gaseous and particulate phases, enabling long-range atmospheric transport before deposition onto soils, vegetation, and aquatic environments [4,5,7,17]. These physicochemical properties account for their persistence, widespread distribution, and accumulation in environmental compartments, resulting in extensive environmental contamination [18]. Although hydrophobic, PAHs can contaminate groundwater, particularly in urbanized and industrialized areas. Benzo[a]pyrene, for example, readily penetrates soil and may subsequently reach groundwater or adjacent surface waters [4].

PAHs enter aquifers through several pathways, including the slow dissolution of contaminated materials, infiltration from polluted soils, and transport mediated by dissolved organic matter and colloidal particles [19,20]. Organic-rich soils frequently act as long-term reservoirs, gradually releasing PAHs into groundwater, particularly under acidic or anaerobic conditions [21]. Historical industrial activities represent the principal sources of groundwater contamination, including former gasworks, coking plants, petroleum refineries [22], wood-treatment facilities using creosote [23,24], contaminated landfills and polluted soils [21], and infiltration of contaminated surface waters into aquifers [25]. In addition, tar and creosote residues may persist below the water table for decades as secondary contamination sources. Low-molecular-weight PAHs, especially naphthalene and phenanthrene, are the compounds most frequently detected in groundwater. Their mobility may be further enhanced through interactions with humic substances and organic colloids, resulting in groundwater concentrations up to 20–50 times higher than those predicted solely from their aqueous solubility [22].

Because of their persistence, bioaccumulation potential, toxicity, and carcinogenicity, PAHs are among the most intensively regulated classes of organic contaminants. Within the European Union, eight PAHs (BaP, DahA, BaA, Chry, BjF, BbF, BkF, and BeP) are regulated under the REACH Regulation (Registration, Evaluation, Authorisation and Restriction of Chemicals) [26], whereas the U.S. Environmental Protection Agency (EPA) has designated sixteen PAHs as priority pollutants for environmental monitoring and risk assessment [27,28].

Human exposure to PAHs occurs primarily through ingestion, inhalation, and dermal contact [18,28,29]. Ingestion represents one of the major exposure pathways for the general population through the consumption of thermally processed foods (e.g., grilled, smoked, baked, fried, and charred products), contaminated cereals, and polluted drinking water [18]. Owing to their pronounced lipophilicity, PAHs are readily absorbed through the gastrointestinal tract [30]. Inhalation exposure occurs mainly in urban and industrial environments through fine particulate matter (PM_2.5_ and PM_10_), to which PAHs are readily adsorbed. Major emission sources include road traffic, industrial activities, residential heating, and cigarette smoke [18]. Dermal exposure is particularly relevant in occupational settings, such as the coke industry and facilities involved in the production or handling of tar, asphalt, and creosote, but may also occur through contact with contaminated water, soil, dust, and other polluted materials [23,31].

Following absorption, PAHs undergo extensive hepatic biotransformation, generating highly reactive metabolites, including epoxides, dihydrodiols, phenols, quinones, and related derivatives, which are capable of forming covalent adducts with DNA and other cellular macromolecules [17,18,30]. Their binding to plasma proteins, particularly albumin, facilitates systemic distribution to various organs [18]. These metabolites induce oxidative stress [8], chronic inflammation, and disturbances in cellular homeostasis [30], thereby contributing to mutagenesis and carcinogenesis.

Most biologically active PAHs contain between two and six fused aromatic rings. Several of the sixteen EPA priority PAHs have been classified by the International Agency for Research on Cancer (IARC) as carcinogenic, probably carcinogenic, or possibly carcinogenic to humans [32], with benzo[a]pyrene (BaP) serving as the reference compound for carcinogenic risk assessment. In addition to their carcinogenic potential [2,3,4,8,9], PAH exposure has been associated with chronic respiratory diseases, cardiovascular disorders, endocrine disruption, impaired liver function, reproductive toxicity, adverse neurodevelopmental outcomes, and digestive tract cancers [1,4,18,30,32]. Prenatal exposure has been linked to reduced birth weight and impaired neurocognitive development [4], whereas phenanthrene exposure has also been associated with hyperuricemia [11]. Acute and chronic dermal exposure may cause irritant dermatitis, photosensitization, precancerous skin lesions, and skin cancer, while compounds such as acenaphthene and pyrene have been associated with skin irritation and other adverse dermatological effects [7,13,17,32].

Despite the environmental and public health relevance of PAHs, studies investigating their occurrence, distribution, sources, and associated environmental and human health risks in groundwater remain relatively scarce, particularly in Europe [20,33,34,35]. Most investigations have focused on contaminated industrial sites or specific hydrogeological settings rather than providing comprehensive regional assessments of groundwater quality.

The occurrence of PAHs in groundwater has been documented in several European countries. For example, investigations conducted at tar-contaminated sites in Germany (Stuttgart, Wülknitz, Castrop-Rauxel, and Lünen) reported naphthalene concentrations of up to 8071.2 μg/L, highlighting the long-term persistence of PAHs in aquifer systems [33]. In Italy, Montuori et al. [20] detected several PAHs, including benzo[g,h,i]perylene, phenanthrene, and naphthalene, in groundwater from the Campania Plain, whereas Riccardi et al. [34] identified eighteen PAHs, ranging from naphthalene to coronene, in groundwater collected from petroleum storage facilities located in northern, central, and southern Italy. Similarly, Llamas et al. [35] investigated organic contaminants in groundwater from the Guadiaro River watershed (southern Spain), reporting pyrene concentrations between 0.001 and 0.015 μg/L. Collectively, these studies demonstrate that PAH occurrence in groundwater is strongly influenced by historical industrial activities, petroleum contamination, land use, and local hydrogeological conditions. However, most of these investigations were performed at contaminated sites and therefore provide limited information on groundwater quality in rural aquifers serving as drinking water resources.

Compared with other European countries, information regarding the occurrence, distribution, and environmental significance of PAHs in Romanian environmental compartments remains limited [36,37,38,39,40,41,42]. Existing studies have mainly focused on sediments, surface waters, marine ecosystems, fish, and drinking water, whereas groundwater has received considerably less attention. In sediments collected from the Begej International Canal (Romania–Serbia), dibenz[a,h]anthracene was identified as the predominant PAH and considered a potential threat to aquatic biota [36]. Ciucure et al. [37] investigated the occurrence, sources, and ecological risks of PAHs in surface waters and sediments from reservoirs along the middle and lower reaches of the Olt River, reporting total PAH concentrations ranging from 1.3 to 46.2 ng/L in water and from 1.78 to 614.04 μg/kg in sediments. Mihaly et al. [38] determined PAH concentrations in scales, gills, liver, and muscle tissues of four farmed fish species from eastern Romania and proposed a rapid approach for evaluating potential human health risks associated with fish consumption. Maldonado et al. [39] reported that PAH contamination in surface waters of the western Black Sea was more pronounced in coastal and estuarine areas, reflecting predominantly petrogenic sources. In rural communities from eastern Romania, Dirtu et al. [40] detected benzo[a]pyrene concentrations of up to 21.78 ng/L in drinking water, while total PAH concentrations ranged from 5.79 to 96.31 ng/L.

Despite these investigations, groundwater contamination by PAHs has received very limited attention in Romania [41,43,44]. Existing studies have been conducted near urban landfills in northern Romania [43], the Bucharest area [41], as well as adjacent to a former petroleum products storage facility in Titu [44]. For example, one study has evaluated PAHs in groundwater affected by leachate migration from the Vidra municipal landfill near Bucharest [41]. Fluorene, phenanthrene, anthracene, fluoranthene, and pyrene were detected at concentrations ranging from 0.0010 to 0.0065 μg/L, whereas all remaining target PAHs were below the analytical detection limits. Overall, available evidence indicates that information regarding the occurrence, distribution, sources, environmental and potential human health risks of PAHs in Romanian groundwater remains fragmentary [36,37,38,39,40,41,42,43,44].

A comprehensive assessment of PAHs occurrence in groundwater requires not only the determination of contaminant concentrations but also the identification of their potential sources and the evaluation of the associated environmental and human health risks. Integrating these complementary approaches provides a robust framework for evaluating groundwater quality and supports the development of effective monitoring and management strategies. To address this knowledge gap, the present study presents the first integrated assessment of PAHs occurrence in groundwater from the central–eastern Romanian Plain, based on the determination of the sixteen EPA priority PAHs. Specifically, the study aimed to (i) determine the occurrence and concentrations of PAHs in groundwater, (ii) identify their potential sources using diagnostic molecular ratios supported by multivariate statistical analysis, (iii) assess the associated environmental risks, and (iv) evaluate the potential non-carcinogenic and carcinogenic health risks related to groundwater consumption. The findings provide baseline data for future groundwater quality monitoring, support evidence-based environmental management, and contribute to a better understanding of PAH occurrence and associated risks in Romanian groundwater.

## 2. Materials and Methods

### 2.1. Sampling and Study Area

**Figure 1:**
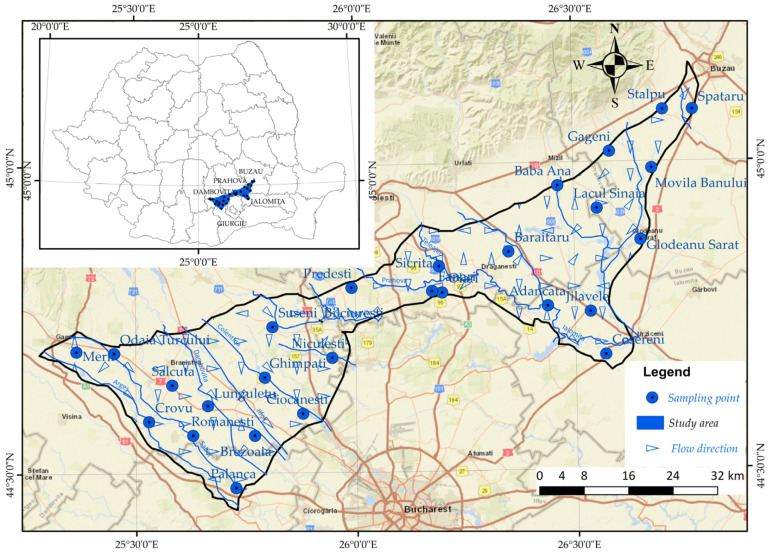
Location of the Titu-Sarata Plain: groundwater flow direction [45,47] and distribution of sampling sites.

The Titu-Sarata Plain, located in the central–eastern part of the Romanian Plain (Figure 1), is a typical subsidence plain, characterized by distinctive morphohydrological features. The region has a temperate continental climate, with a mean annual air temperature of 10–11 °C, and an average annual precipitation of 550–600 mm. Although precipitation is relatively uniformly distributed throughout the year, the overall rainfall amount is sufficiently low to favour the development of steppe vegetation.

The hydrographic network is dominated by a northwest–southeast drainage pattern and includes the Arges, Dambovita, Ialomita, and Cricovul Sarat rivers, together with several smaller tributaries. In addition, several natural and artificial lakes contribute to the ecological diversity of the region. Groundwater occurs at relatively shallow depths (5–10 m), exerting a strong influence on soil moisture conditions and agricultural land use.

The landscape is dominated by agricultural land, primarily arable fields, interspersed with secondary steppe grasslands and fragmented oak forests. This land-cover pattern is reflected in the spatial distribution of soils, with Chernozems and halomorphic soils prevailing in the eastern part of the plain, whereas the Luvisols and Cambisols are predominant in the western part [45]. The region has a population density of approximately 130 inhabitants/km^2^, exceeding the national average [45,46]. Population distribution is heterogeneous, with higher densities in the southwestern sector and lower densities in the eastern part of the plain. Apart from the towns of Titu, Bolintin Vale, and Racari, the area is predominantly rural, with agriculture representing the principal economic activity. Wheat and maize are the dominant crops, while several localities including Brezoaia, Lunguletu, Potlogi, and Poiana, are well known for intensive potatoes and vegetables cultivation.

Shallow groundwater is located in porous-permeable deposits belonging to the Upper Pleistocene and Holocene, consisting of alternating sands and gravels. The granulometry of these deposits varies from fine sands interspersed with clays in the southern area to coarse gravel and boulders towards the contact area with the piedmont hills in the northwest. Hydraulic conductivity is heterogeneous, being influenced by the granulometry of the deposits, and varies between 2.9 × 10^−2^ cm/s and 4 × 10^−2^ cm/s. In the fine deposits consisting of sandy-clayey silts, water circulation is reduced, which favors intense evapotranspiration processes and increased mineralization. Shallow groundwater is recharged by precipitation (in the central and southern parts), and locally, a balance exists between the aquifer and the hydrographic network in small areas. The general direction of groundwater flow is NW–SE [45,47].

Groundwater samples were collected from 27 rural localities distributed throughout the Titu–Sarata Plain. The sampling sites were selected to ensure representative spatial coverage of the study area [48] (Figure 1).

The map was produced using ArcGIS 10.8 (Esri, Redlands, CA, USA), while the digital terrain model (DTM) was generated from Shuttle Radar Topography Mission (SRTM) data obtained from the U.S. Geological Survey EarthExplorer platform (https://earthexplorer.usgs.gov/) (accesed on 15 June 2026).

Sampling was carried out during a single campaign in October 2023. Water samples were collected from public dug wells with groundwater depths ranging from 2 to 20 m. In small rural settlements where there is no water supply network, groundwater from dug wells is used by residents for drinking water, domestic consumption, or irrigation. In settlements with a drinking water supply system, the groundwater source is represented by medium and deep aquifers.

Sample collection, handling, transportation, and storage were performed in accordance with EPA regulations [48,49]. Water was collected in sterile amber glass bottles previously rinsed with the sampled water and transported to the laboratory under refrigerated conditions (<4 °C), avoiding freezing prior to analysis.

### 2.2. Analytical Determination of PAHs by UHPLC-FLD

All solvents and reagents used for PAHs analysis, including n-hexane, acetonitrile, ultrapure water, and anhydrous sodium sulfate, were of HPLC grade (Merck, Darmstadt, Germany). The certified reference material PAH-Mix 18 (10 mg/L in acetonitrile), containing the sixteen U.S. EPA priority PAHs, was purchased from LGC Standards (Teddington Middlesex, London, United Kingdom).

PAHs were determined according to the international standard [50] which describes the determination of 15 PAHs in water by high-performance liquid chromatography with fluorescence detection (HPLC-FLD) following liquid–liquid extraction. To determine total PAH concentrations, water samples were extracted without prior filtration, thereby including both dissolved and particle-associated PAH fractions in the analysis. Briefly, 1 L of water sample was transferred into a separatory funnel and extracted three consecutive times with 25 mL of n-hexane by vigorous shaking for 5 min during each extraction. The organic extracts were passed successively through anhydrous sodium sulfate to remove traces of water contents and subsequently combined and concentrated to approximately 5 mL using a vacuum solvent concentrator (Multivapor P-6 Professional, Büchi, Sweden). The extract was then evaporated to dryness under a gentle stream of nitrogen using a TurboVap LV system (Biotage, Uppsala, Sweden). Finally, the residue was reconstituted in 1.0 mL of acetonitrile, filtered through a 0.45 μm membrane filter, and subjected to HPLC analysis.

Quantitative determination of fifteen of the sixteen U.S. EPA priority PAHs (Nap, Ace, Flu, Phe, Ant, Flt, Pyr, BaA, Chry, BbF, BkF, BaP, DahA, BghiP, and IcdP) was performed using a Dionex UHPLC system coupled with a fluorescence detector (UHPLC-FLD; Thermo Fisher Scientific, Bremen, Germany). Acenaphthylene was not included because it does not exhibit native fluorescence and therefore cannot be determined by fluorescence detection. Chromatographic separation was performed on a Hypersil Green PAH analytical column (250 × 4.6 mm, 5 μm particle size) fitted with a matching guard column (10 × 4 mm, 5 μm) from Thermo Fisher Scientific. Gradient elution was performed using water (mobile phase A) and acetonitrile (mobile phase B). The optimized gradient program (0 min 50% B, 35 min 100% B, 55 min 100% B, 60 min 50% B, 65 min 50% B), together with time-programmed excitation (Ex) and emission (Em) wavelengths, was adopted from our previously validated method [37]. The fluorescence detector was operated at the following wavelength pairs: 0.1–19.0 min, Ex 224 nm, and Em 330 nm; 19.0–25.0 min, Ex 275 nm, and Em 350 nm; 25.0–29.0 min, Ex 290 nm, and Em 430 nm; 29.0–34.0 min, Ex 270 nm, and Em 430 nm; 34.0–39.5 min, Ex 260 nm, and Em 420 nm; 39.5–52.5 min, Ex 290 nm, and Em 430 nm; and 52.5–60.0 min, Ex 305 nm, and Em 480 nm. The injection volume was 25 μL.

Quantification was performed using the external standard method with calibration solutions in the range of 0.1–25 μg/L for each compound. Method validation and quality assurance/quality control (QA/QC) procedures were carried out in accordance with the international standard [51] and our previously validated analytical protocol [37]. Method detection limits (LODs) ranged from 0.06 to 0.42 ng/L, while limits of quantification (LOQs) ranged from 0.15 to 0.99 ng/L. Method precision varied between 0.3 and 4.4%, and recovery experiments performed on spiked water samples using the certified reference material PAH-Mix 18 yielded recoveries between 86.9 and 108.9%, indicating negligible matrix effects and satisfactory method performance. Method blanks and quality control (QC) standard solutions were analyzed with each batch of 10 samples as part of the quality assurance/quality control (QA/QC) protocol to assess potential contamination and verify instrument performance. No target analytes were detected in the method blanks above the method detection limits, confirming the absence of significant laboratory contamination. The concentrations measured in the QC standard solutions were within the established control limits for each individual PAH, demonstrating satisfactory analytical performance and method stability throughout the analysis. All samples underwent duplicate measurements, and the results are reported as mean values expressed in ng/L.

### 2.3. Diagnostic Ratio Method

Potential sources of PAHs in groundwater were identified using the molecular diagnostic ratio approach, one of the most widely applied methods for distinguishing between petrogenic and pyrogenic inputs [37,52,53,54,55,56,57]. The method is based on comparing the concentration ratios of selected pairs of PAH isomers measured in the samples with characteristic threshold values reported for different contamination sources (Table 1) [37,52,53,54,55,56,57].

The diagnostic ratios are based on the different thermodynamic stability of PAH isomers formed during low- and high-temperature processes. Low-temperature petrogenic processes preferentially generate less thermodynamically stable isomers, whereas high-temperature pyrogenic processes favour the formation of more thermodynamically stable isomers [57]. Low-molecular-weight PAHs (LMW-PAHs) comprise compounds containing two or three aromatic rings, whereas high-molecular-weight PAHs (HMW-PAHs) contain four to six aromatic rings [37,52,58].

Because individual diagnostic ratios may occasionally provide inconsistent source assignments owing to environmental weathering and transformation processes [52,56,57], an integrated Total Index (TI) was also calculated according to Equation (1), providing a more robust classification of PAH sources:

Total Index = Ant/Ant+Phe/0.1 + Flt/Flt+Pyr/0.4 + BaA/BaA+Chry/0.2 + IcdP/IcdP+BghiP/0.5(1)

### 2.4. Assessment of Individual and Total Environmental Risk Coefficients

The ecological risks associated with the occurrence of PAHs in groundwater were evaluated using the individual risk quotient (RQ_i_) and the cumulative risk quotient (RQ_tot_) approaches [46,59,60]. The individual risk quotient for each PAH was calculated as the ratio between its measured concentration in groundwater (C_wi_) and the corresponding predicted no-effect concentration (PNEC), according to Equation (2):

RQ_i_ = C_wi_/PNEC, (2)

The PNEC values were selected from the NORMAN Ecotoxicology Database [46,59,61,62], using experimentally derived values whenever available and QSAR-predicted values when experimental data were unavailable.

The cumulative ecological risk for each sampling site was expressed as the total risk quotient (RQ_tot_), calculated as the sum of the individual risk quotients of all detected PAHs (Equation (3)):

RQ_tot_ = ΣRQ_i_, (3)

The ecological risk was classified according to both individual (RQ_i_) and cumulative (RQ_tot_) risk quotients as follows: values <0.1 indicate negligible ecological risk, values between 0.1 and 1 indicate low ecological risk, values between 1 and 10 indicate moderate ecological risk, and values >10 indicate high ecological risk [52,59].

The additive risk model provides an integrated assessment of the potential ecological effects associated with the simultaneous occurrence of multiple contaminants in groundwater. However, this approach assumes concentration additivity and does not account for potential unpredictable synergistic or antagonistic interactions among co-occurring contaminants, which may influence the overall ecotoxicological response [59,61].

### 2.5. Human Health Risk Assessment

#### 2.5.1. Hazard Quotient and Hazard Index

The potential non-carcinogenic health risks associated with PAH exposure through groundwater ingestion were evaluated using the Hazard Quotient (HQ) and Hazard Index (HI) approaches [63,64,65,66,67,68,69,70]. The Hazard Quotient for each individual PAH (HQ_i_) was calculated according to Equation (4):

HQ_i_ = C_wi_ × IR × ED × EF/BW × AT × RfD_i_, (4)

where C_wi_ is the concentration of the individual PAH in groundwater (mg/L); IR is the daily water ingestion rate (L/day); EF is the exposure frequency (days/year); ED is the exposure duration (years); AT is the averaging time (days); BW is the average body weight (kg) for each age group of consumers; RfD_i_ is the oral Reference Dose for the corresponding PAH (mg/kg/day).

The cumulative non-carcinogenic risk associated with simultaneous exposure to multiple PAHs was estimated using Hazard Index (HI) calculated as the sum of the individual Hazard Quotients (Equation (5)) [71]:

HI = ΣHQ_i_, (5)

According to U.S. EPA risk assessment guidelines [64,70], HQ or HI values <1 indicate no appreciable non-carcinogenic risk, values between 1 and 10 indicate potential non-carcinogenic health effects, whereas values >10 indicate a high non-carcinogenic risk for the exposed population.

#### 2.5.2. Incremental Lifetime Cancer Risks Assessment

For mixtures of PAHs, the carcinogenic potency of individual compounds was expressed relative to benzo[a]pyrene (BaP), which is widely used as the reference carcinogen for PAH risk assessment [72]. Because toxicological data are not available for all PAHs, individual concentrations were converted into benzo[a]pyrene equivalent concentrations (BaP_eq_) using potency equivalency factors (PEFs), according to Equation (6) [72]:

BaP_eq_ = ∑(C_wi_ × PEF_i_), (6)

where C_wi_ is the individual concentration of PAH in water samples (µg/L) and PEF_i_ is the potency equivalency factor of the ith individual contaminant.

The carcinogenic risk associated with oral exposure to PAHs through groundwater ingestion was estimated using the Incremental Lifetime Cancer Risk (ILCR) model, calculated according to Equation (7) [46]:

ILCR_ingestion_ = BaP_eq_ × IR × ED × EF × CSF/BW × AT, (7)

where IR is the daily water ingestion rate (L/day); EF is the exposure frequency (days/year); ED is the exposure duration (years); AT is the averaging time (days); CSF is cancer slope factor (mg/kg/day)^−1^; BW is the average body weight (kg) for each age group of consumers.

According to the U.S. EPA risk assessment framework [65,73,74,75], ILCR values below 10^−6^ are generally considered to represent negligible or acceptable carcinogenic risk, values between 10^−6^ and 10^−4^ indicate a potential carcinogenic risk, whereas values exceeding 10^−4^ are considered to represent a high carcinogenic risk.

## 3. Results

### 3.1. Level of PAHs in Water Samples

Thirteen of the sixteen U.S. EPA priority PAHs were detected in the analyzed groundwater samples (Table 2). Dibenzo[a,h]anthracene (DahA) and indeno [1,2,3-cd]pyrene (IcdP) were not detected in any sample.

Total PAH concentrations ranged from 2.56 to 11.2 ng/L, indicating generally low levels of groundwater contamination throughout the study area. Benzo[a]pyrene (BaP), the reference carcinogenic PAH regulated in drinking water, was detected at concentrations ranging from <LOD to 0.08 ng/L, while the sum of the four regulated PAHs (Σ4PAHs) ranged from <LOD to 1.81 ng/L.

All measured concentrations were substantially below the maximum permissible limits established by the European Drinking Water Directive [76,77,78,79] and Romanian legislation (0.010 μg/L for BaP and 0.10 μg/L for Σ4PAHs) [80], indicating that all groundwater samples complied with the current regulatory standards.

Among the detected compounds, phenanthrene was consistently the predominant PAH, reaching a maximum concentration of 5.53 ng/L, followed by naphthalene (2.35 ng/L), fluorene (1.71 ng/L), fluoranthene (1.47 ng/L), and pyrene (1.43 ng/L). In contrast, high-molecular-weight PAHs, including benzo[k]fluoranthene, benzo[a]pyrene, chrysene, and benzo[b]fluoranthene, were detected only at trace concentrations or were absent from many sampling locations. The predominance of low- and medium-molecular-weight PAHs suggests a greater environmental mobility of these compounds and is consistent with their higher aqueous solubility compared with higher-ring PAHs.

**Figure 2:**
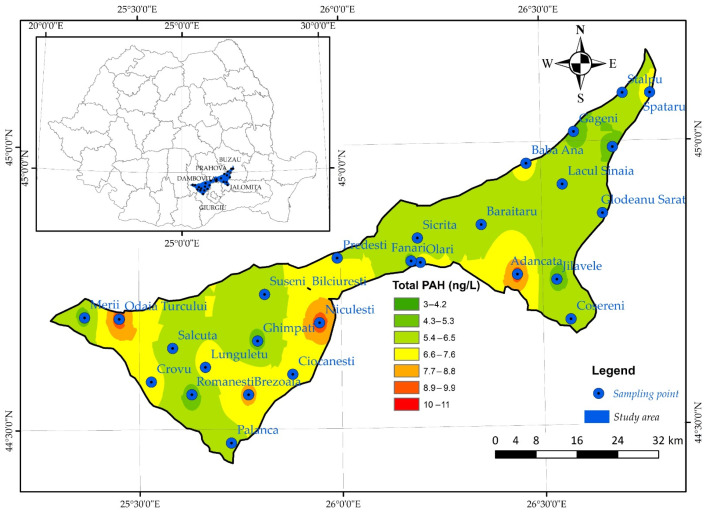
Spatial distribution of total PAH concentrations (ng/L) in the analyzed water samples.

The spatial distribution of total PAHs is presented in Figure 2. The highest total PAH concentration (11.2 ng/L) was recorded in Adancata, Niculesti, and Odaia Turcului, whereas lower concentrations were observed in the remaining localities. According to the classification proposed by Moisés Canle [52], all groundwater samples fall within the category of low PAH contamination (0–100 ng/L), confirming the overall good chemical quality of the investigated groundwater with respect to PAHs.

**Figure 3:**
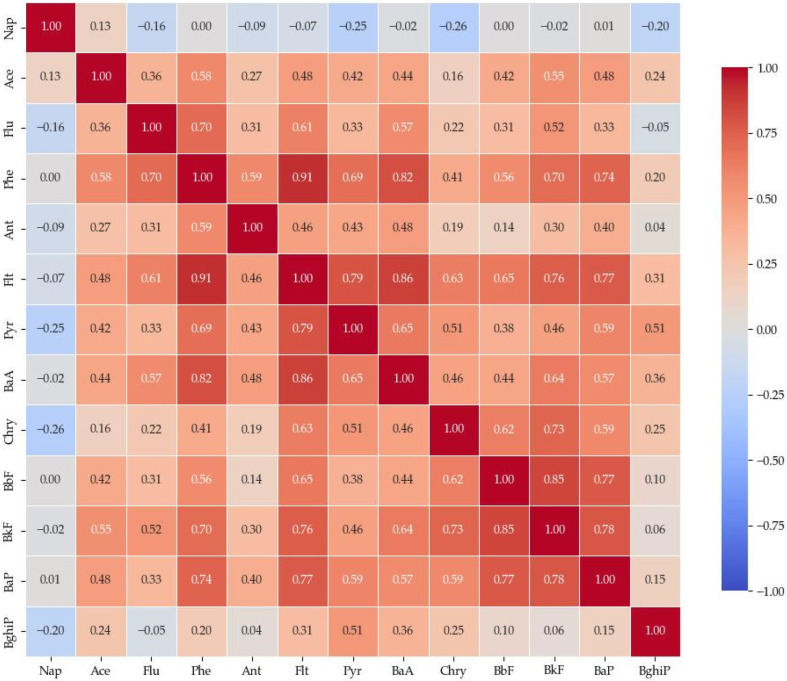
Correlation matrix for the PAHs determined in the groundwater samples.

The correlation matrix (Figure 3), performed using the Python-based Library scikit-learn, version 1.8.0 (The scikit-learn Developers, Open-Source Community, International), revealed several significant positive relationships among the detected PAHs, suggesting common emission sources and similar environmental behaviour within the aquifer. The strongest correlations (r > 0.80) were observed for Phe–Flt (r = 0.91), Flt–BaA (r = 0.86), BbF–BkF (r = 0.85), and Phe–BaA (r = 0.82). Additional strong associations were found for BkF–BaP (r = 0.78) and Flt–BaP (r = 0.77). These relationships indicate that these compounds tend to co-occur in groundwater and likely share common contamination sources, transport pathways, and attenuation processes, reflecting their similar physicochemical properties.

In contrast, benzo[g,h,i]perylene exhibited predominantly weak correlations with the remaining PAHs, except for moderate relationships with pyrene (r = 0.51) and benzo[a]anthracene (r = 0.36), suggesting differences in source contributions or environmental behaviour. Naphthalene also displayed a distinct correlation pattern, showing weak or negative correlations with most higher-molecular-weight PAHs, particularly chrysene (r = −0.26), and pyrene (r = −0.25). This behaviour is consistent with the higher volatility, greater aqueous solubility, and lower molecular weight of naphthalene, which influence its transport and degradation differently from those of heavier PAHs. Overall, the correlation analysis indicates that the distribution of PAHs in groundwater is controlled by both common contamination sources and compound-specific physicochemical properties.

### 3.2. Identification of PAHs Contamination Sources

To identify the potential sources of PAHs in groundwater, the molecular diagnostic ratios Ant/(Ant+Phe), Flt/(Flt+Pyr), BaA/(BaA+Chry), and the integrated Total Index (TI) were calculated for all sampling locations (Table 3). The combined interpretation of these diagnostic indicators revealed that PAH contamination in the investigated groundwater originates predominantly from pyrogenic and mixed pyrogenic–petrogenic sources, while purely petrogenic signatures were identified only in a limited number of localities.

Based on the diagnostic ratios and Total Index values, groundwater from Baba Ana, Cosereni, Niculesti, Palanca, Sinaia Lac, Suseni Bilciuresti, and Salcuta exhibited predominantly pyrogenic signatures. In Baba Ana, Cosereni, Suseni Bilciuresti, and Salcuta, the PAH profile is consistent with the combustion of fossil and liquid fuels, whereas in Niculesti, Palanca, and Sinaia Lac, the diagnostic ratios suggest a greater contribution from biomass combustion, including wood and agricultural residues. Petrogenic signatures were identified only in Ghimpati and Romanesti, while the remaining localities showed mixed contamination patterns with a predominance of pyrogenic inputs.

The predominance of pyrogenic signatures is consistent with the socio-economic characteristics of the investigated rural area. Because centralized heating systems are generally absent, households rely primarily on wood, coal, and liquid fuels for domestic heating. In addition, the open burning of agricultural residues and household plant waste, although prohibited by Romanian legislation, remains a common practice and represents an important local source of atmospheric PAHs. This interpretation is supported by reports from the Dambovița County Environmental Guard, which documented 520 vegetation and agricultural waste fires in 2024 [81] and a further 289 similar events in 2025 [82].

The diagnostic ratio values indicate that the sources of PAH pollution in the localities of Ghimpati and Romanesti are petrogenic. Mixed pollution sources, predominantly pyrogenic, are identified in the localities of Adancata, Brezoaia, Baraitaru, Ciocanesti, Crovu, Fanari, Gageni, Glodeanu Sarat, Lunguletu, Movila Banului, Odaia Turcului, Olarii Vechi, Predesti, Stalpu, Spataru, and Sicrita; and mixed, predominantly petrogenic, in the localities of Jilavele and Merii. Petrogenic PAHs are typically associated with crude oil and refined petroleum products released through accidental spills, leaking underground storage tanks, or illegal fuel extraction activities. Several such incidents have been reported over recent years along the oil transportation infrastructure operated by Conpet S.A. and within oil-producing areas of Prahova and Dambovita counties. Documented cases in Gageni [83], Brezoaia [84], Spataru, and Stalpu provide plausible local sources for the petrogenic contribution identified in some groundwater samples.

**Figure 4:**
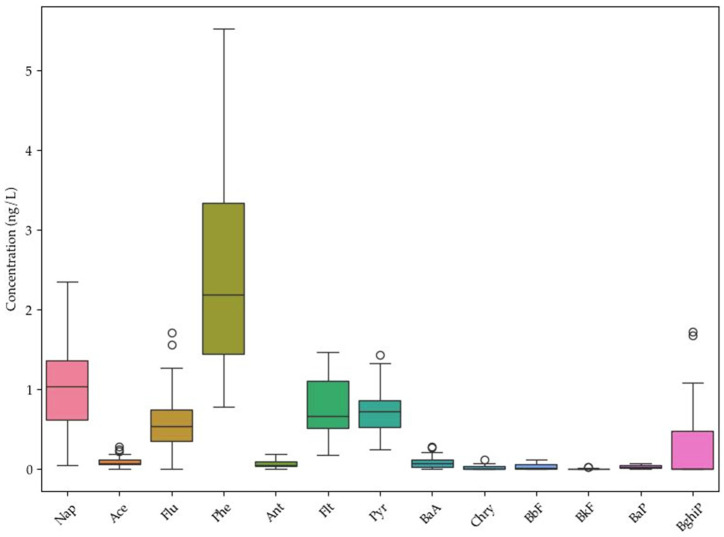
Boxplot representation of the distribution of PAHs concentrations in groundwater samples.

**Figure 5:**
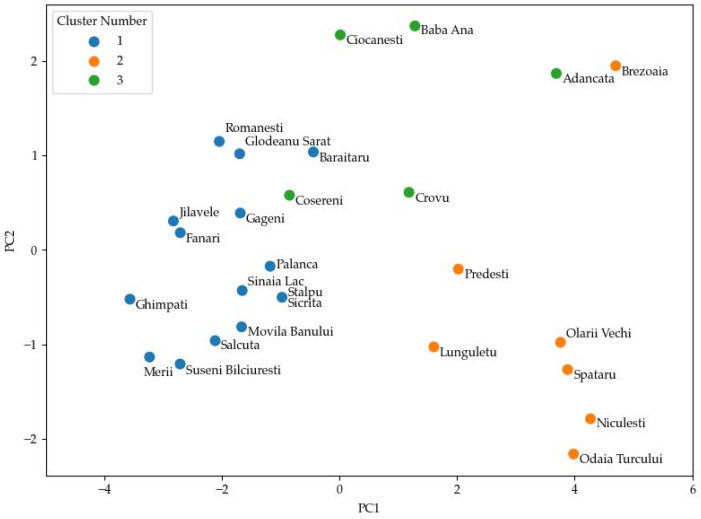
PCA projection of the sampling locations based on PAH concentrations determined in groundwater samples.

To further evaluate the consistency of the source apportionment results, multivariate statistical analysis was performed using boxplot visualization (Figure 4) and Principal Component Analysis (PCA), performed with the Python-based Library scikit-learn, version 1.8.0 (Figure 5). For PCA, concentrations below the LOD were substituted with LOD/2, a commonly applied approach for handling left-censored environmental data, to allow the inclusion of all samples in the multivariate statistical analysis. These complementary approaches provided an independent assessment of the variability of PAH concentrations and the similarity among sampling locations.

The boxplot presented in Figure 4 shows a heterogeneous distribution of PAHs concentrations, with Phe and Nap standing out due to higher concentrations in central areas, while high-molecular-weight compounds such as BaA, Chry, BbF, BkF, and BaP generally exhibit low concentrations. The marked variability observed for Phe, as well as the moderate dispersion of Flu, Flt, and Pyr, suggests significant spatial differences between sampling localities and an uneven distribution of sources or transport processes within the aquifer. The presence of extreme values for certain compounds indicates localized point sources, and the relative predominance of light and intermediate PAHs may reflect higher mobility in the subsurface and a possible petrogenic or mixed influence on the contamination profile.

The results of the PCA analysis, highlighting the distribution of sampling locations of groundwater samples, are presented in Figure 5, and reveal the existence of three distinct groups, based on their PAHs concentration profiles. An uneven distribution of localities within the PCA space is evident, with a clear grouping of some sampling localities—such as Romanesti, Glodeanu Sarat, and Baraitaru—in cluster 1, the separation of the sampling localities Brezoaia, Olarii Vechi, Spataru, Niculesti, and Odaia Turcului in Cluster 2, and the distinct positioning of the sampling localities Ciocanesti, Baba Ana, Adancata, Crovu, and Cosereni in Cluster 3, which reflects the existence of distinct PAH compositional profiles in the investigated groundwater. The clustering pattern supports the diagnostic ratio analysis, indicating that groundwater contamination is controlled by multiple local emission sources and that the relative contribution of low- and high-molecular-weight PAHs varies considerably across the investigated area. The identified clusters exhibit only a partial geographical pattern, indicating that the distribution of PAHs is not controlled solely by the spatial location of the sampling sites. Cluster 1 is mainly concentrated in the central and eastern sectors of the Titu-Sarata Plain, whereas cluster 2 is predominantly associated with the western and southwestern sectors, although it also includes several eastern localities. Cluster 3 is mainly distributed in the central and northeastern part of the studied area. This spatial arrangement suggests that, besides geographical proximity, the heterogeneous hydrogeological conditions of the shallow aquifer, such as lithological variability, differences in hydraulic conductivity and the regional northwest-southeast groundwater flow, together with local anthropogenic activities, play an important role in shaping the identified PAHs.

### 3.3. Ecological Risk Assessment

The ecological risk associated with PAHs in groundwater was evaluated using individual risk quotients (RQ_i_) and cumulative risk quotients (RQ_tot_). Overall, the results indicate negligible ecological risks for most detected PAHs, whereas fluoranthene (Flt) was identified as the principal contributor to the overall ecological risk. Depending on the sampling location, the cumulative ecological risk ranged from low to moderate, with RQ_tot_ values between 0.2 and 2.3 (Table 4).

The lowest freshwater predicted no-effect concentrations (PNECs) available in the NORMAN Ecotoxicology Database [62] were used for ecological risk assessment. The adopted PNEC values (μg/L) were 2.0 for naphthalene (Nap), 3.7 for acenaphthene (Ace), 0.25 for fluorene (Flu), 0.00076 for fluoranthene (Flt), 0.0046 for pyrene (Pyr), 0.07 for chrysene (Chry), 0.017 for benzo[b]fluoranthene (BbF), 0.017 for benzo[k]fluoranthene (BkF), 0.27 for benzo[a]pyrene (BaP), and 0.0082 for benzo[g,h,i]perylene (BghiP) [62]. Because of their relatively high PNEC values and the low concentrations measured in groundwater, naphthalene, acenaphthene, fluorene, chrysene, benzo[b]fluoranthene, benzo[k]fluoranthene, and benzo[a]pyrene exhibited RQ values below 0.01 in all samples and therefore made only a negligible contribution to the overall ecological risk.

As shown in Table 4, fluoranthene was the dominant contributor to the ecological risk, with individual RQ values ranging from 0.2 to 1.9. Moderate ecological risk associated with fluoranthene (RQ > 1) was identified in eleven sampling locations, whereas pyrene and benzo[g,h,i]perylene exhibited considerably lower RQ values, generally below 0.2. Consequently, variations in the cumulative ecological risk among sampling sites were controlled primarily by differences in fluoranthene concentrations.

Based on the cumulative risk quotient, most groundwater samples were classified as presenting either low (RQ_tot_ = 0.1–1) or moderate (RQ_tot_ = 1–10) ecological risk, while no location reached the threshold for high ecological risk. The highest cumulative risk (RQ_tot_ = 2.3) was observed in Brezoaia, followed by Adancata (2.2), Odaia Turcului (1.8), Niculesti (1.7), and Olarii Vechi (1.6). Although these values indicate only moderate ecological risk, they suggest that fluoranthene should be considered a priority compound for future groundwater monitoring within the investigated area.

### 3.4. Human Health Risks Assessment

Although the concentrations of individual PAHs measured in groundwater were well below the maximum permissible limits for drinking water, simultaneous exposure to multiple compounds may result in cumulative non-carcinogenic and carcinogenic health effects. Therefore, human health risks associated with groundwater ingestion were evaluated separately for infants, children, and adults using age-specific exposure parameters (Table 5) [46,63,67,69]:

#### 3.4.1. Non-Carcinogenic Risk Assessment

The oral reference dose (RfD) values used for the calculation of the individual Hazard Quotients (HQ_i_) were 2 × 10^−2^ mg/kg/day for naphthalene (Nap) [7], 2 × 10^−1^ mg/kg/day for acenaphthene (Ace) [10], 8 × 10^−4^ mg/kg/day for fluorene (Flu) [12], 1 mg/kg/day for anthracene (Ant) [8], 4 × 10^−2^ mg/kg/day for fluoranthene (Flt) [2], 3 × 10^−2^ mg/kg/day for pyrene (Pyr) [13], and 3 × 10^−4^ mg/kg/day for benzo[a]pyrene (BaP) [85].

The calculated HQ_i_ values for individual PAHs are presented in Supplementary Data (Tables S1–S3). For all age groups, fluorene and benzo[a]pyrene exhibited the highest individual HQ values whereas anthracene and acenaphthene showed the lowest contributions to the overall non-carcinogenic risk. Although infants consistently showed higher HQ values than children and adults because of their lower body weight and higher water intake relative to body mass, all calculated HQ values remained several orders of magnitude below the threshold value of 1.

The cumulative non-carcinogenic risk, expressed as the Hazard Index (HI), ranged from 1.0 × 10^−5^ to 2.6 × 10^−4^ for infants, from 0.8 × 10^−5^ to 1.17 × 10^−4^ for children, and from 0.4 × 10^−5^ to 6.6 × 10^−5^ for adults (Table 6). The highest HI values were recorded in Adancata and Niculesti for all three age groups, whereas the lowest values were observed in Cosereni, Suseni Bilciuresti, and Romanesti. Nevertheless, all HI values were substantially lower than the safety threshold (HI = 1), indicating that groundwater consumption does not pose appreciable non-carcinogenic health risks for infants, children, or adults in the investigated area. Similar findings have previously been reported for groundwater affected by the Vidra landfill (Romania) [41].

#### 3.4.2. Carcinogenic Risk Assessment

The carcinogenic potency of the detected PAHs was expressed as benzo[a]pyrene equivalent concentrations (BaPeq) using the potency equivalency factors (PEFs) recommended by the Guidance for Calculating Benzo[a]pyrene Equivalents for Cancer Evaluations of Polycyclic Aromatic Hydrocarbons [72]. The applied PEF values were 1 for benzo[a]pyrene, 0.1 for benzo[a]anthracene, benzo[b]fluoranthene, benzo[k]fluoranthene, and indeno [1,2,3-cd]pyrene, 0.01 for chrysene, and 2.4 for dibenzo[a,h]anthracene. The calculated BaP concentrations together with the corresponding Incremental Lifetime Cancer Risk (ILCR) values for infants, children, and adults are presented in Table 7.

BaPeq concentrations ranged from 3.0 × 10^−8^ to 1.1 × 10^−7^ mg/L. The highest BaPeq values were observed in Odaia Turcului and Spataru (1.1 × 10^−7^ mg/L), followed by Brezoaia and Niculesti (9.0 × 10^−8^ mg/L), whereas no carcinogenic PAHs were detected in Ghimpati, Jilavele, and Merii. Consequently, no ILCR values were calculated for these localities.

The calculated ILCR values ranged from 5.7 × 10^−9^ to 2.2 × 10^−8^ for infants, from 2.7 × 10^−9^ to 1.0 × 10^−8^ for children, and from 1.5 × 10^−9^ to 6.1 × 10^−9^ for adults (Table 7). For all sampling locations and age groups, ILCR values remained well below the generally accepted carcinogenic risk threshold of 10^−6^, indicating that groundwater ingestion does not pose an appreciable lifetime carcinogenic risk to the exposed population. As observed for the non-carcinogenic risk assessment, infants exhibited the highest estimated carcinogenic risk because of their lower body weight and higher water intake relative to body mass, whereas adults showed the lowest ILCR values.

## 4. Discussion

Total PAH concentrations measured in groundwater from the Titu–Sarata Plain ranged from 2.56 to 11.29 ng/L, with a mean value of 6.28 ng/L. These levels were lower than those reported for groundwater from the Campania Plain, Italy, where total PAH concentrations ranged from 0.65 to 34.1 ng/L [20]. They were also substantially lower than concentrations reported in groundwater from a coal-mining area in northern Anhui Province, China (15.04–449.13 ng/L) [58], and from tube wells in Belém, Brazil (20.4–91.1 µg/L) [86]. Moreover, the concentrations observed in the present study were several orders of magnitude lower than those reported near the Ruseifa landfill, Jordan (7.1–12.6 mg/L; mean: 9.1 mg/L) [87]. The very high levels recorded in Jordan are due to the direct impact of leachate from the landfill.

Although our study focuses on a rural region with no obvious sources of pollution, the individual PAH concentrations were generally comparable to those reported by Balint et al. [41] for groundwater samples collected near the Vidra landfill in Romania, which represents a local source of contamination. For example, phenanthrene concentrations ranged from 0.79 to 5.53 ng/L in the present study, compared with 1.4–6.5 ng/L in the Vidra area. Similar concentration ranges were also observed for fluorene (<LOD–1.71 ng/L vs. 1.0–3.7 ng/L) and fluoranthene (0.18–1.47 ng/L vs. 1.0–1.1 ng/L). In contrast, lower concentrations were recorded in the Titu–Sarata Plain for anthracene (<LOD–0.19 ng/L vs. 1.0–1.3 ng/L) and pyrene (0.25–1.43 ng/L vs. 3.0–3.2 ng/L), suggesting a lower contribution of some higher-molecular-weight PAHs compared with the landfill-affected groundwater system.

The ecological risk assessment showed that most individual PAHs posed negligible risk, particularly Nap, Ace, Flu, Chry, BbF, BkF, and BaP. Pyrene was associated with low ecological risk, whereas fluoranthene represented the main contributor to ecological risk, with values ranging from low to moderate. For BghiP, negligible risk was observed in most localities, although low-risk levels were identified in Glodeanu Sarat, Crovu, Ciocanesti, Brezoaia, Baba Ana, and Adancata. Based on the cumulative risk quotient, low ecological risk (RQ_tot_ = 0.2–0.9) was identified in Baraitaru, Cosereni, Gageni, Ghimpati, Jilavele, Merii, Movila Banului, Palanca, Romanesti, Sinaia Lac, Suseni Bilciuresti, and Salcuta. Moderate ecological risk (RQ_tot_ = 1.0–2.3) was observed in Adancata, Baba Ana, Brezoaia, Ciocanesti, Crovu, Lunguletu, Niculesti, Odaia Turcului, Olarii Vechi, Predesti, Stalpu, Spataru, and Sicrita. These values are comparable to those reported by Jiang et al. [58] for groundwater from a coal-mining area in northern Anhui Province, China.

The human health risk assessment indicated that exposure to PAHs through groundwater ingestion does not pose appreciable non-carcinogenic risk for any of the investigated population groups. In all localities, both HQ_i_ and HI values were several orders of magnitude below the threshold value of 1, indicating negligible non-carcinogenic risk for infants, children, and adults. Similarly, the calculated ILCR values remained well below the commonly accepted carcinogenic risk threshold. Although the ILCR values obtained in the present study were higher than those reported by Montuori et al. [20] for the Campania Plain, Italy (7.3 × 10^−20^–4.96 × 10^−19^), they were still extremely low, ranging from 5.7 × 10^−9^ to 2.2 × 10^−8^ for infants, 2.7 × 10^−9^ to 1.0 × 10^−8^ for children, and 1.5 × 10^−9^ to 6.1 × 10^−9^ for adults. These results indicate negligible lifetime carcinogenic risk associated with groundwater ingestion in the investigated area.

The findings highlight the importance of continued groundwater quality monitoring in the Titu–Sarata Plain. Previous evidence of pesticide contamination in the same region [46], together with the occurrence of PAHs reported in the present study, indicates that groundwater quality may be influenced by multiple classes of organic contaminants. Future investigations should therefore expand the analytical scope to include other contaminant groups, such as heavy metals and additional organic micropollutants, in order to provide a more comprehensive assessment of groundwater quality and potential cumulative risks in this rural area.

A limitation of this study is that groundwater was sampled during a single campaign, preventing the evaluation of seasonal variability and temporal changes in PAHs concentrations.

## 5. Conclusions

This study provides the first integrated assessment of polycyclic aromatic hydrocarbons (PAHs) in groundwater from the Titu–Sarata Plain, combining chemical determination with source apportionment, ecological risk assessment, and human health risk evaluation. Thirteen of the sixteen U.S. EPA priority PAHs were detected, while overall PAH concentrations remained low (2.56–11.29 ng/L) and complied with current European drinking water quality standards.

The combined interpretation of molecular diagnostic ratios, correlation analysis, and principal component analysis demonstrated that groundwater PAHs originate predominantly from pyrogenic and mixed pyrogenic–petrogenic sources. The observed spatial heterogeneity reflects the influence of multiple local contamination sources together with differences in the environmental behaviour and mobility of individual PAHs within the aquifer. Low-molecular-weight PAHs were generally more abundant than higher-molecular-weight compounds, consistent with their greater mobility in groundwater.

Ecological risk assessment indicated predominantly low ecological risk throughout the investigated area, although moderate cumulative ecological risk was identified at several sampling locations. Fluoranthene was the principal contributor to the ecological risk, whereas most of the remaining PAHs exhibited negligible individual ecological risks.

The human health risk assessment demonstrated that groundwater ingestion does not pose appreciable non-carcinogenic or carcinogenic risks for infants, children, or adults. Both Hazard Index (HI) and Incremental Lifetime Cancer Risk (ILCR) values remained well below the internationally accepted safety thresholds for all investigated groundwater samples.

Although the current results indicate a favourable groundwater quality with respect to PAHs, the occurrence of these contaminants, together with previously reported pesticide residues in the same region, highlights the importance of integrated groundwater quality monitoring. Future investigations should extend the range of monitored contaminants to include additional organic micropollutants and heavy metals, as well as repeated sampling campaigns to assess seasonal variability and temporal chances, thereby providing a more comprehensive assessment of groundwater quality and supporting sustainable groundwater resource management in rural areas.

## Figures and Tables

**Table 1:** Threshold values of PAH diagnostic ratios used for source identification [37,52,53,54,55,56,57].

Diagnostic Ratio	Petrogenic Source	Pyrogenic Source	Intermediate Range	Interpretation
Ant/(Ant+Phe)	<0.10	>0.10	-	-
Flt/(Flt+Pyr)	<0.40	>0.50	0.40–0.50	petroleum combustion
BaA/(BaA+Chry)	<0.20	>0.35	0.20–0.35	mixed combustion
IcdP/(IcdP+Bghi)	<0.20	>0.5	0.20–0.50	petroleum combustion
LMW-PAHs/HMW-PAHs	>1	<1	-	-
Total index	<4	>4	-	-

**Table 2:** Concentrations (ng/L) of identified PAHs in water samples.

Location	Nap	Ace	Flu	Phe	Ant	Flt	Pyr	BaA	Chry	BbF	BkF	BaP	BghiP
Adancata	0.58	0.12	1.71	4.81	0.1	1.47	1.15	0.29	0.05	nd	0.01	0.03	0.89
Baba Ana	0.45	nd	0.67	3.58	0.18	0.96	1.0	0.19	0.04	nd	nd	0.04	0.78
Brezoaia	0.66	0.25	0.45	3.43	0.05	1.38	1.43	0.22	0.08	0.11	0.02	0.06	1.68
Baraitaru	0.4	0.03	0.95	2.49	0.05	0.68	0.82	0.08	nd	0.05	nd	0.03	0.5
Ciocanesti	0.79	0.07	0.52	2.31	0.08	0.81	1.33	0.04	0.03	nd	nd	0.03	0.96
Cosereni	1.25	0.13	nd	2.4	0.19	0.66	0.74	0.07	nd	nd	nd	0.02	0.43
Crovu	1.56	0.19	0.26	2.71	0.06	0.8	0.69	0.12	0.04	0.06	0.01	0.05	1.73
Fanari	0.66	nd	0.33	1.09	0.03	0.5	0.46	0.01	nd	nd	nd	0.03	nd
Gageni	0.63	0.08	0.6	1.44	0.05	0.5	0.53	0.04	nd	nd	nd	0.04	0.31
Glodeanu Sarat	0.82	nd	0.27	1.63	nd	0.65	0.57	0.03	nd	0.06	nd	0.03	1.09
Ghimpati	1.13	0.13	0.33	0.79	nd	0.18	0.28	nd	nd	nd	nd	nd	0.16
Jilavele	0.49	0.05	0.75	1.08	nd	0.51	0.41	nd	0.02	nd	nd	nd	nd
Lunguletu	1.41	0.09	0.61	3.74	0.04	1.12	0.84	0.06	0.07	0.07	0.01	0.06	nd
Merii	1.54	0.08	0.73	1.16	0.03	0.31	0.25	nd	nd	nd	nd	nd	nd
Movila Banului	1.25	0.06	0.41	1.5	0.04	0.55	0.53	0.03	0.01	0.06	nd	0.03	nd
Niculesti	1.23	0.29	1.56	5.53	0.14	1.2	1.06	0.1	nd	0.09	0.02	0.07	nd
Odaia Turcului	1.82	0.23	1.05	5.05	0.1	1.3	0.87	0.27	nd	0.08	0.02	0.07	nd
Olarii Vechi	0.62	0.1	1.27	3.86	0.11	1.16	0.74	0.2	0.07	0.12	0.03	0.05	nd
Predesti	0.62	0.06	0.5	2.69	0.08	1.06	0.88	0.11	0.08	0.1	0.02	0.05	nd
Palanca	0.94	0.06	0.45	2.07	0.13	0.62	0.53	0.04	0.01	0.03	nd	0.03	nd
Romanesti	0.05	0.12	nd	1.13	0.04	0.38	0.78	0.01	0.02	nd	nd	0.03	nd
Stalpu	1.38	0.09	0.69	1.99	0.05	0.78	0.72	0.07	nd	0.04	nd	0.02	0.09
Spataru	1.15	0.1	0.84	3.06	0.1	1.12	0.87	0.12	0.12	0.12	0.03	0.08	0.11
Sinaia Lac	1.31	0.07	0.55	1.58	0.08	0.63	0.6	0.04	nd	nd	nd	0.03	nd
Suseni Bilciuresti	2.35	nd *	nd	1.47	nd	0.55	0.64	0.07	nd	nd	nd	0.02	nd
Salcuta	1.8	0.08	0.5	1.2	0.07	0.48	0.53	0.07	nd	nd	nd	0.02	nd
Sicrita	1.31	0.03	0.16	2.26	0.06	0.76	0.67	0.07	0.01	0.05	nd	0.04	nd

* nd = not detected.

**Table 3:** Values of the PAH diagnostic ratios calculated for the analyzed water samples.

Location	Ant/Ant+Phe	Flt/Flt+Pyr	BaA/BaA+Chry	Total Index
Adancata	0.06	0.56	0.85	6.30
Baba Ana	0.16	0.49	0.83	6.93
Brezoaia	0.03	0.49	0.73	5.24
Baraitaru	0.07	0.45	1.00	6.82
Ciocanesti	0.09	0.38	0.57	4.70
Cosereni	0.22	0.47	1.00	8.41
Crovu	0.07	0.54	0.75	5.79
Fanari	0.06	0.52	1.00	6.87
Gageni	0.09	0.49	1.00	7.12
Glodeanu Sarat	na	0.53	1.00	6.33
Ghimpati	na	0.39	na	0.98
Jilavele	* na	0.55	na	1.39
Lunguletu	0.03	0.57	0.46	4.08
Merii	0.09	0.55	na	2.27
Movila Banului	0.07	0.51	0.75	5.70
Niculesti	0.10	0.53	1.00	7.37
Odaia Turcului	0.07	0.60	1.00	7.21
Olarii Vechi	0.09	0.61	0.74	6.10
Predesti	0.07	0.55	0.58	4.96
Palanca	0.17	0.54	0.80	7.08
Romanesti	0.10	0.33	0.33	3.44
Stalpu	0.06	0.52	1.00	6.90
Spataru	0.08	0.56	0.50	4.73
Sinaia Lac	0.11	0.51	1.00	7.41
Suseni Bilciuresti	na	0.46	1.00	6.16
Salcuta	0.13	0.48	1.00	7.46
Sicrita	0.07	0.53	0.88	6.44

* na = not applicable.

**Table 4:** Values of individual environmental risk coefficients (RQ) and total risk coefficients (RQ_tot_) associated with PAHs.

Location	RQ Flt	RQ Pyr	RQ BghiP	RQ_tot_
Adancata	1.9	0.2	0.1	2.2
Baba Ana	1.2	0.2	0.1	1.5
Brezoaia	1.8	0.3	0.2	2.3
Baraitaru	0.8	0.1	<0.01	0.9
Ciocanesti	1.0	0.2	0.1	1.3
Cosereni	0.8	0.1	<0.01	0.9
Crovu	1.0	0.1	0.2	1.3
Fanari	0.6	0.1	* na	0.7
Gageni	0.6	0.1	<0.01	0.7
Glodeanu Sarat	0.8	0.1	0.1	1.0
Ghimpati	0.2	0.0	<0.01	0.2
Jilavele	0.6	0.0	na	0.6
Lunguletu	1.4	0.1	na	1.5
Merii	0.4	0.0	na	0.4
Movila Banului	0.7	0.1	na	0.8
Niculesti	1.5	0.2	na	1.7
Odaia Turcului	1.7	0.1	na	1.8
Olarii Vechi	1.5	0.1	na	1.6
Predesti	1.3	0.1	na	1.4
Palanca	0.8	0.1	na	0.9
Romanesti	0.5	0.1	na	0.6
Stalpu	1.0	0.1	<0.01	1.1
Spataru	1.4	0.1	<0.01	1.5
Sinaia Lac	0.8	0.1	na	0.9
Suseni Bilciuresti	0.7	0.1	na	0.8
Salcuta	0.6	0.1	na	0.7
Sicrita	1.0	0.1	na	1.1

* na = not applicable.

**Table 5:** The parameters values used for the health risk assessment for each age group.

Parameter	Infants	Children	Adults
Age	2	11	70
IR (L/day)	0.75	1	2
ED (year)	2	11	70
EF (days/year)	365	365	365
AT (ED × EF) (days)	2 × 365	11 × 365	70 × 365
BW (kg)	7	20	70

**Table 6:** HI values for infants, children, and adults.

Location	HI Infants	HI Children	HI Adults
Adancata	2.5 × 10^−4^	11.7 × 10^−5^	6.6 × 10^−5^
Baba Ana	1.1 × 10^−4^	5.2 × 10^−5^	3.0 × 10^−5^
Brezoaia	0.9 × 10^−4^	4.3 × 10^−5^	2.5 × 10^−5^
Baraitaru	1.4 × 10^−4^	6.7 × 10^−5^	3.8 × 10^−5^
Ciocanesti	0.9 × 10^−4^	4.2 × 10^−5^	2.4 × 10^−5^
Cosereni	0.2 × 10^−4^	0.8 × 10^−5^	0.4 × 10^−5^
Crovu	0.7 × 10^−4^	3.0 × 10^−5^	1.7 × 10^−5^
Fanari	0.6 × 10^−4^	2.8 × 10^−5^	1.6 × 10^−5^
Gageni	1.0 × 10^−4^	4.7 × 10^−5^	2.7 × 10^−5^
Glodeanu Sarat	0.6 × 10^−4^	2.5 × 10^−5^	1.4 × 10^−5^
Ghimpati	0.5 × 10^−4^	2.4 × 10^−5^	1.3 × 10^−5^
Jilavele	1.1 × 10^−4^	4.9 × 10^−5^	2.8 × 10^−5^
Lunguletu	1.2 × 10^−4^	5.4 × 10^−5^	3.1 × 10^−5^
Merii	1.1 × 10^−4^	5.0 × 10^−5^	2.8 × 10^−5^
Movila Banului	0.8 × 10^−4^	3.5 × 10^−5^	2.0 × 10^−5^
Niculesti	2.6 × 10^−4^	11.6 × 10^−5^	6.6 × 10^−5^
Odaia Turcului	1.8 × 10^−4^	8.5 × 10^−5^	4.8 × 10^−5^
Olarii Vechi	2.0 × 10^−4^	9.2 × 10^−5^	5.2 × 10^−5^
Predesti	0.9 × 10^−4^	4.3 × 10^−5^	2.5 × 10^−5^
Palanca	0.8 × 10^−4^	3.7 × 10^−5^	2.1 × 10^−5^
Romanesti	0.1 × 10^−4^	6.9 × 10^−5^	0.3 × 10^−5^
Stalpu	1.1 × 10^−4^	5.2 × 10^−5^	2.9 × 10^−5^
Spataru	1.5 × 10^−4^	7.1 × 10^−5^	4.0 × 10^−5^
Sinaia Lac	1.0 × 10^−4^	4.4 × 10^−5^	2.5 × 10^−5^
Suseni Bilciuresti	0.2 × 10^−4^	1.0 × 10^−5^	0.6 × 10^−5^
Salcuta	0.9 × 10^−4^	4.0 × 10^−5^	2.3 × 10^−5^
Sicrita	0.5 × 10^−4^	2.2 × 10^−5^	1.2 × 10^−5^

**Table 7:** BaPeq (mg/L) and ILCR values for the three age groups.

Location	BaPeq mg/L	ILCR Infants	ILCR Children	ILCR Adults
Adancata	6.0 × 10^−8^	1.2 × 10^−8^	5.9 × 10^−9^	3.4 × 10^−9^
Baba Ana	6.0 × 10^−8^	1.2 × 10^−8^	5.8 × 10^−9^	3.3 × 10^−9^
Brezoaia	9.0 × 10^−8^	2.0 × 10^−8^	9.4 × 10^−9^	5.3 × 10^−9^
Baraitaru	4.0 × 10^−8^	9.2 × 10^−9^	4.3 × 10^−9^	2.4 × 10^−9^
Ciocanesti	3.0 × 10^−8^	7.2 × 10^−9^	3.3 × 10^−9^	1.9 × 10^−9^
Cosereni	3.0 × 10^−8^	5.7 × 10^−9^	2.7 × 10^−9^	1.5 × 10^−9^
Crovu	7.0 × 10^−8^	1.4 × 10^−8^	6.8 × 10^−9^	3.9 × 10^−9^
Fanari	3.0 × 10^−8^	6.6 × 10^−9^	3.1 × 10^−9^	1.7 × 10^−9^
Gageni	4.0 × 10^−8^	9.4 × 10^−9^	4.4 × 10^−9^	2.5 × 10^−9^
Glodeanu Sarat	4.0 × 10^−8^	8.3 × 10^−9^	3.9 × 10^−9^	2.2 × 10^−9^
Ghimpati	* na	na	na	na
Jilavele	na	na	na	na
Lunguletu	7.0 × 10^−8^	1.5 × 10^−8^	7.3 × 10^−9^	4.1 × 10^−9^
Merii	na	na	na	na
Movila Banului	4.0 × 10^−8^	8.3 × 10^−9^	3.8 × 10^−9^	2.2 × 10^−9^
Niculesti	9.0 × 10^−8^	1.9 × 10^−8^	9.1 × 10^−9^	5.2 × 10^−9^
Odaia Turcului	11.0 × 10^−8^	2.2 × 10^−8^	1.0 × 10^−8^	6.1 × 10^−9^
Olarii Vechi	8.0 × 10^−8^	1.8 × 10^−8^	8.4 × 10^−9^	4.8 × 10^−9^
Predesti	7.0 × 10^−8^	1.5 × 10^−8^	7.2 × 10^−9^	4.1 × 10^−9^
Palanca	4.0 × 10^−8^	7.9 × 10^−9^	3.6 × 10^−9^	2.1 × 10^−9^
Romanesti	3.0 × 10^−8^	6.6 × 10^−9^	3.0 × 10^−9^	1.7 × 10^−9^
Stalpu	3.0 × 10^−8^	6.6 × 10^−9^	3.1 × 10^−9^	1.7 × 10^−9^
Spataru	11.0 × 10^−8^	2.2 × 10^−8^	1.0 × 10^−8^	6.0 × 10^−9^
Sinaia Lac	3.0 × 10^−8^	7.2 × 10^−9^	3.4 × 10^−9^	1.9 × 10^−9^
Suseni Bilciuresti	3.0 × 10^−8^	5.7 × 10^−9^	2.7 × 10^−9^	1.5 × 10^−9^
Salcuta	3.0 × 10^−8^	5.7 × 10^−9^	2.7 × 10^−9^	1.5 × 10^−9^
Sicrita	5.0 × 10^−8^	1.1 × 10^−8^	5.1 × 10^−9^	2.9 × 10^−9^

* na = not applicable.

## Data Availability

The raw data supporting the conclusions of this article will be made available by the authors on request.

## Supplementary Materials

The following supporting information can be downloaded at https://www.mdpi.com/article/10.3390/toxics14070638/s1, Table S1: The Hazard Quotient (HQ_i_) values individual PAHs for infants; Table S2: The Hazard Quotient (HQ_i_) values individual PAHs for children; Table S3: The Hazard Quotient (HQ_i_) values individual PAHs for adults.

## Abbreviations

The following abbreviations are used in this manuscript:

PAHs	Polycyclic Aromatic Hydrocarbons
LMW-PAHs	Low-Molecular-Weight PAHs
HMW-PAHs	High Polycyclic Aromatic Hydrocarbons
IARC	The International Agency for Research on Cancer
USEPA	The U.S. Environmental Protection Agency
ECHA	The European Chemicals Agency
REACH	Registration, Evaluation, Authorisation and Restriction of Chemicals
ATDSR	The Agency for Toxic Substances and Disease Registry
UHPLC-FLD	High-Performance Liquid Chromatography coupled with Fluorescence Detection
PNEC	The lowest predicted no-effect concentrations
RQi	Individual risk quotients
RQtot	Cumulative risk quotients
RfD	Oral reference dose
HQi	Individual Hazard Quotients
HI	Hazard Index
PEFs	Potency equivalency factors
BaPeq	Benzo[a]pyrene equivalent concentrations
ILCR	Incremental Lifetime Cancer Risk
Nap	Naphthalene
Acy	Acenaphthylene
Ace	Acenaphthene
Flu	Fluorene
Phe	Phenanthrene
Ant	Anthracene
Flt	Fluoranthene
Pyr	Pyrene
BaA	Benz[a]anthracene
Chry	Chrysene
BbF	Benzo[b]fluoranthene
BkF	Benzo[k]fluoranthene
BaP	Benzo[a]pyrene
IcdP	Indeno [1,2,3-cd]pyrene
DahA	Dibenz[a,h]anthracene
BghiP	Benzo[g,h,i]perylene
∑4PAH	The sum of the concentrations BbF, BkF, BghiP and IcdP ompounds
QA	The quality assurance
QC	The quality control
