# Supplementary material for: Occurrence of Contaminants in Groundwater from the Central–Northeastern Part of the Romanian Plain and the Associated Risk Assessment
Source: Toxics. 2026 Jul 21;14(7):638. doi: 10.3390/toxics14070638 (PMC13417692; doi:10.3390/toxics14070638)
Supplement: Supplementary file 1 [file toxics-14-00638-s001.zip › toxics-4437101-supplementary.pdf]

Supplementary Data

Occurrence of Contaminants in Groundwater from the Central–Northeastern Part of the Romanian Plain and the Associated Risk Assessment

Crinela Dumitrescu [1,*], Claudia Stihi [1,2,*], Roxana Elena Ionete [3], Elisabeta-Irina Geană [3], Corina Teodora Ciucure [3] and Petre Brețcan [4]

[1] Valahia University of Targoviste, Faculty of Sciences and Arts, 13 Aleea Sinaia St., 130004 Targoviste, Romania; crinela.dumitrescu@valahia.ro (C.D.); claudia.stihi@valahia.ro (C.S.)
[2] Academy of Romanian Scientists, 3 Ilfov St., 050044 Bucharest, Romania; claudia.stihi@valahia.ro (C.S.)
[3] National Research and Development Institute for Cryogenic and Isotopic Technologies, Ramnicu-Valcea. Romania; roxana.ionete@icsi.ro (R.E.I.); irina.geana@icsi.ro (E.I.G.); corina.ciucure@icsi.ro (C.T.C.)
[4] Valahia University of Targoviste, Faculty of Humanities, 35 Stancu Ion St., 130105 Targoviste, Romania; petre.bretcan@valahia.ro (P.B.)
* Correspondence: crinela.dumitrescu@valahia.ro (C.D); claudia.stihi@valahia.ro (C.S);

Table S1. The Hazard Quotient (HQ_i) values individual PAHs for infants

Location	HQ Nap	HQ Ace	HQ Flu	HQ Ant	HQ Flt	HQ Pyr	HQ BaP
Adancata	$3.11x10^{-6}$	$6.43\ x10^{-8}$	$22.9\ x10^{-5}$	$1.07\ x10^{-8}$	$3.94x10^{-6}$	$4.11x10^{-6}$	$1.07\ x10^{-5}$
Baba Ana	$2.41x10^{-6}$	na	$8.97\ x10^{-5}$	$1.93\ x10^{-8}$	2.57x10-6	$3.57x10^{-6}$	$1.43\ x10^{-5}$
Brezoaia	$3.54x10^{-6}$	$1.34\ x10^{-7}$	$6.03\ x10^{-5}$	$5.36\ x10^{-9}$	$3.7x10^{-6}$	$5.11\ x10^{-6}$	$2.14\ x10^{-5}$
Baraitaru	$2.14x10^{-6}$	$1.61\ x10^{-8}$	$12.7\ x10^{-5}$	$5.36\ x10^{-9}$	$1.82x10^{-6}$	$2.93\ x10^{-6}$	$1.07\ x10^{-5}$
Ciocanesti	$4.23x10^{-6}$	$3.75\ x10^{-8}$	$6.96\ x10^{-5}$	$8.57\ x10^{-9}$	$2.17x10^{-6}$	$4.75\ x10^{-6}$	$1.07\ x10^{-5}$
Cosereni	$6.7x10^{-6}$	$6.96\ x10^{-8}$	na	$2.04\ x10^{-8}$	$1.77x10^{-6}$	$2.64\ x10^{-6}$	$7.14\ x10^{-6}$
Crovu	$8.36x10^{-6}$	$1.02\ x10^{-7}$	$3.48\ x10^{-5}$	$6.43\ x10^{-9}$	$2.14x10^{-6}$	$2.46\ x10^{-6}$	$1.79\ x10^{-5}$
Fanari	$3.54x10^{-6}$	na	$4.42\ x10^{-5}$	$3.21\ x10^{-9}$	$1.34x10^{-6}$	$1.64\ x10^{-6}$	$1.07\ x10^{-5}$
Gageni	$3.38x10^{-6}$	$4.29\ x10^{-8}$	$8.04\ x10^{-5}$	$5.36\ x10^{-9}$	$1.34x10^{-6}$	$1.89\ x10^{-6}$	$1.43\ x10^{-5}$
Glodeanu Sarat	$4.39x10^{-6}$	na	$3.62\ x10^{-5}$	na	$1.74x10^{-6}$	$2.04\ x10^{-6}$	$1.07\ x10^{-5}$
Ghimpati	$6.05x10^{-6}$	$6.96\ x10^{-8}$	$4.42\ x10^{-5}$	na	$4.82\ x10^{-7}$	$0.1\ x10^{-5}$	na
Jilavele	$2.63x10^{-6}$	$2.68\ x10^{-8}$	$1\ x10^{-4}$	na	$1.37x10^{-6}$	$1.46\ x10^{-6}$	na
Lunguletu	$7.55x10^{-6}$	$4.82\ x10^{-8}$	$8.17\ x10^{-5}$	$4.29\ x10^{-9}$	$0.3\ x10^{-5}$	$0.3\ x10^{-5}$	$2.14\ x10^{-5}$
Merii	$8.25x10^{-6}$	$4.29\ x10^{-8}$	$9.78\ x10^{-5}$	$3.21\ x10^{-9}$	$8.3\ x10^{-7}$	$8.93\ x10^{-7}$	na
Movila Banului	$6.7x10^{-6}$	$3.21\ x10^{-8}$	$5.49\ x10^{-5}$	$4.29\ x10^{-9}$	$1.47x10^{-6}$	$1.89\ x10^{-6}$	$1.07\ x10^{-5}$
Niculesti	$6.59x10^{-6}$	$1.55\ x10^{-7}$	$20.9\ x10^{-5}$	$1.5\ x10^{-8}$	$3.21x10^{-6}$	$3.79\ x10^{-6}$	$2.5\ x10^{-5}$
Odaia Turcului	$9.75x10^{-6}$	$1.23\ x10^{-7}$	$14.1\ x10^{-5}$	$1.07\ x10^{-8}$	$3.48x10^{-6}$	$3.11\ x10^{-6}$	$2.5\ x10^{-5}$
Olarii Vechi	$3.32x10^{-6}$	$5.36\ x10^{-8}$	$17\ x10^{-5}$	$1.18\ x10^{-8}$	$3.11x10^{-6}$	$2.64\ x10^{-6}$	$1.79\ x10^{-5}$
Predesti	$3.32x10^{-6}$	$3.21\ x10^{-8}$	$6.7\ x10^{-5}$	$8.57\ x10^{-9}$	$2.84x10^{-6}$	$3.14\ x10^{-6}$	$1.79\ x10^{-5}$
Palanca	$5.04x10^{-6}$	$3.21\ x10^{-8}$	$6.03\ x10^{-5}$	$1.39\ x10^{-8}$	$1.66x10^{-6}$	$1.89\ x10^{-6}$	$1.07\ x10^{-5}$
Romanesti	$2.68\ x10^{-7}$	$6.43\ x10^{-8}$	na	$4.29\ x10^{-9}$	$1.02x10^{-6}$	$2.79\ x10^{-6}$	$1.07\ x10^{-5}$
Stalpu	$7.39x10^{-6}$	$4.82\ x10^{-8}$	$9.24\ x10^{-5}$	$5.36\ x10^{-9}$	$2.09x10^{-6}$	$2.57\ x10^{-6}$	$7.14\ x10^{-6}$
Spataru	$6.16x10^{-6}$	$5.36\ x10^{-8}$	$11.3\ x10^{-5}$	$1.07\ x10^{-8}$	$0.3\ x10^{-5}$	$3.11\ x10^{-6}$	$2.86\ x10^{-5}$
Sinaia Lac	$7.02x10^{-6}$	$3.75\ x10^{-8}$	$7.37\ x10^{-5}$	$8.57\ x10^{-9}$	$1.69x10^{-6}$	$2.14\ x10^{-6}$	$1.07\ x10^{-5}$
Suseni Bilciuresti	$1.26\ x10^{-5}$	na	na	na	$1.47x10^{-6}$	$2.29\ x10^{-6}$	$7.14\ x10^{-6}$
Salcuta	$9.64x10^{-6}$	$4.29\ x10^{-8}$	$6.7\ x10^{-5}$	$7.5\ x10^{-9}$	$1.29x10^{-6}$	$1.89x10^{-6}$	$7.14\ x10^{-6}$
Sicrita	$7.02x10^{-6}$	$1.61\ x10^{-8}$	$2.14\ x10^{-5}$	$6.43\ x10^{-9}$	$2.04x10^{-6}$	$2.39x10^{-6}$	$1.43\ x10^{-5}$

* na = not applicable

Table S2. The Hazard Quotient (HQ_i) values individual PAHs for children

Location	HQ Nap	HQ Ace	HQ Flu	HQ Ant	HQ Flt	HQ Pyr	HQ BaP
Adancata	1.45 x10^{-6}	3 x10^{-8}	10.7 x10^{-5}	5 x10^{-9}	1.84 x10^{-6}	1.92 x10^{-6}	5 x10^{-6}
Baba Ana	1.13 x10^{-6}	na*	4.19 x10^{-5}	9 x10^{-9}	1.2 x10^{-6}	1.67 x10^{-6}	6.67 x10^{-6}
Brezoaia	1.65 x10^{-6}	6.25 x10^{-8}	2.81 x10^{-5}	2.5 x10^{-9}	1.73 x10^{-6}	2.38 x10^{-6}	1 x10^{-5}
Baraitaru	1 x10^{-6}	7.5 x10^{-9}	5.94 x10^{-5}	2.5 x10^{-9}	8.5 x10^{-7}	1.37 x10^{-6}	5 x10^{-6}
Ciocanesti	1.98 x10^{-6}	1.75 x10^{-8}	3.25 x10^{-5}	4 x10^{-9}	1.01 x10^{-6}	2.22 x10^{-6}	5 x10^{-6}
Cosereni	3.13 x10^{-6}	3.25 x10^{-8}	na	9.5 x10^{-9}	8.25 x10^{-7}	1.23 x10^{-6}	3.33 x10^{-6}
Crovu	3.9 x10^{-6}	4.75 x10^{-8}	1.63 x10^{-5}	3 x10^{-9}	1 x10^{-6}	1.15 x10^{-6}	8.33 x10^{-6}
Fanari	1.65 x10^{-6}	na	2.06 x10^{-5}	1.5 x10^{-9}	6.25 x10^{-7}	7.67 x10^{-7}	5 x10^{-6}
Gageni	1.58 x10^{-6}	2 x10^{-8}	3.75 x10^{-5}	2.5 x10^{-9}	6.25 x10^{-7}	8.83 x10^{-7}	6.67 x10^{-6}
Glodeanu Sarat	2.05 x10^{-6}	na	1.69 x10^{-5}	na	8.13 x10^{-7}	9.5 x10^{-7}	0.5 x10^{-5}
Ghimpati	2.83 x10^{-6}	3.25 x10^{-8}	2.06 x10^{-5}	na	2.25 x10^{-7}	4.67 x10^{-7}	na
Jilavele	1.23 x10^{-6}	1.25 x10^{-8}	4.69 x10^{-5}	na	6.38 x10^{-7}	6.83 x10^{-7}	na
Lunguletu	3.53 x10^{-6}	2.25 x10^{-8}	3.81 x10^{-5}	2 x10^{-9}	1.4 x10^{-6}	1.4 x10^{-6}	1 x10^{-5}
Merii	3.85 x10^{-6}	2 x10^{-8}	4.56 x10^{-5}	1.5 x10^{-9}	3.88 x10^{-7}	4.17 x10^{-7}	na
Movila Banului	3.13 x10^{-6}	1.5 x10^{-8}	2.56 x10^{-5}	2 x10^{-9}	6.88 x10^{-7}	8.83 x10^{-7}	5 x10^{-6}
Niculesti	3.08 x10^{-6}	7.25 x10^{-8}	9.75 x10^{-5}	7 x10^{-9}	1.5 x10^{-6}	1.77 x10^{-6}	1.17 x10^{-5}
Odaia Turcului	4.55 x10^{-6}	5.75 x10^{-8}	6.56 x10^{-5}	5 x10^{-9}	1.63 x10^{-6}	1.45 x10^{-6}	1.17 x10^{-5}
Olarii Vechi	1.55 x10^{-6}	2.5 x10^{-8}	7.94 x10^{-5}	5.5 x10^{-9}	1.45 x10^{-6}	1.23 x10^{-6}	8.33 x10^{-6}
Predesti	1.55 x10^{-6}	1.5 x10^{-8}	3.13 x10^{-5}	4 x10^{-9}	1.33 x10^{-6}	1.47 x10^{-6}	8.33 x10^{-6}
Palanca	2.35 x10^{-6}	1.5 x10^{-8}	2.81 x10^{-5}	6.5 x10^{-9}	7.75 x10^{-7}	8.83 x10^{-7}	5 x10^{-6}
Romanesti	1.25 x10^{-7}	3 x10^{-8}	na	2 x10^{-9}	4.75 x10^{-7}	1.3 x10^{-6}	5 x10^{-6}
Stalpu	3.45 x10^{-6}	2.25 x10^{-8}	4.31 x10^{-5}	2.5 x10^{-9}	9.75 x10^{-7}	1.2 x10^{-6}	3.33 x10^{-6}
Spataru	2.88 x10^{-6}	2.5 x10^{-8}	5.25 x10^{-5}	5 x10^{-9}	1.4 x10^{-6}	1.45 x10^{-6}	1.33 x10^{-5}
Sinaia Lac	3.28 x10^{-6}	1.75 x10^{-8}	3.44 x10^{-5}	4 x10^{-9}	7.88 x10^{-7}	1 x10^{-6}	5 x10^{-6}
Suseni Bilciuresti	5.88 x10^{-6}	na	na	na	6.88 x10^{-7}	1.07 x10^{-6}	3.33 x10^{-6}
Salcuta	4.5 x10^{-6}	2 x10^{-8}	3.13 x10^{-5}	3.5 x10^{-9}	6 x10^{-7}	8.83 x10^{-7}	3.33 x10^{-6}
Sicrita	3.28 x10^{-6}	7.5 x10^{-9}	1 x10^{-5}	3 x10^{-9}	9.5 x10^{-7}	1.12 x10^{-6}	6.67 x10^{-6}

* na = not applicable

Table S3. The Hazard Quotient (HQ_i) values individual PAHs for adults

Location	HQ Nap	HQ Ace	HQ Flu	HQ Ant	HQ Flt	HQ Pyr	HQ BaP
Adancata	8.29 x10^{-7}	1.71 x10^{-8}	6.11 x10^{-5}	2.86 x10^{-9}	1.05 x10^{-6}	1.1 x10^{-6}	2.86 x10^{-6}
Baba Ana	6.43 x10^{-7}	na	2.39 x10^{-5}	5.14 x10^{-9}	6.86 x10^{-7}	9.52 x10^{-7}	3.81 x10^{-6}
Brezoaia	9.43 x10^{-7}	3.57 x10^{-8}	1.61 x10^{-5}	1.43 x10^{-9}	9.86 x10^{-7}	1.36 x10^{-6}	5.71 x10^{-6}
Baraitaru	5.71 x10^{-7}	4.29 x10^{-9}	3.39 x10^{-5}	1.43 x10^{-9}	4.86 x10^{-7}	7.81 x10^{-7}	2.86 x10^{-6}
Ciocanesti	1.13 x10^{-6}	1 x10^{-8}	1.86 x10^{-5}	2.29 x10^{-9}	5.79 x10^{-7}	1.27 x10^{-6}	2.86 x10^{-6}
Cosereni	1.79 x10^{-6}	1.86 x10^{-8}	na	5.43 x10^{-9}	4.71 x10^{-7}	7.05 x10^{-7}	1.9 x10^{-6}
Crovu	2.23 x10^{-6}	2.71 x10^{-8}	9.29 x10^{-6}	1.71 x10^{-9}	5.71 x10^{-7}	6.57 x10^{-7}	4.76 x10^{-6}
Fanari	9.43 x10^{-7}	na	1.18 x10^{-5}	8.57 x10^{-10}	3.57 x10^{-7}	4.38 x10^{-7}	2.86 x10^{-6}
Gageni	9 x10^{-7}	1.14 x10^{-8}	2.14 x10^{-5}	1.43 x10^{-9}	3.57 x10^{-7}	5.05 x10^{-7}	3.81 x10^{-6}
Glodeanu Sarat	1.17 x10^{-6}	na	9.64 x10^{-6}	na	4.64 x10^{-7}	5.43 x10^{-7}	2.86 x10^{-6}
Ghimpati	1.61 x10^{-6}	1.86 x10^{-8}	1.18 x10^{-5}	na	1.29 x10^{-7}	2.67 x10^{-7}	na
Jilavele	7 x10^{-7}	7.14 x10^{-9}	2.68 x10^{-5}	na	3.64 x10^{-7}	3.9 x10^{-7}	na
Lunguletu	2.01 x10^{-6}	1.29 x10^{-8}	2.18 x10^{-5}	1.14 x10^{-9}	8 x10^{-7}	8 x10^{-7}	5.71 x10^{-6}
Merii	2.2 x10^{-6}	1.14 x10^{-8}	2.61 x10^{-5}	8.57 x10^{-10}	2.21 x10^{-7}	2.38 x10^{-7}	na

Location	HQ Nap	HQ Ace	HQ Flu	HQ Ant	HQ Flt	HQ Pyr	HQ BaP
Movila Banului	1.79×10^{-6}	8.57×10^{-9}	1.46×10^{-5}	1.14×10^{-9}	3.93×10^{-7}	5.05×10^{-7}	2.86×10^{-6}
Niculesti	1.76×10^{-6}	4.14×10^{-8}	5.57×10^{-5}	4×10^{-9}	8.57×10^{-7}	1.01×10^{-6}	6.67×10^{-6}
Odaia Turcului	2.6×10^{-6}	3.29×10^{-8}	3.75×10^{-5}	2.86×10^{-9}	9.29×10^{-7}	8.29×10^{-7}	6.67×10^{-6}
Olarii Vechi	8.86×10^{-7}	1.43×10^{-8}	4.54×10^{-5}	3.14×10^{-9}	8.29×10^{-7}	7.05×10^{-7}	4.76×10^{-6}
Predesti	8.86×10^{-7}	8.57×10^{-9}	1.79×10^{-5}	2.29×10^{-9}	7.57×10^{-7}	8.38×10^{-7}	4.76×10^{-6}
Palanca	1.34×10^{-6}	8.57×10^{-9}	1.61×10^{-5}	3.71×10^{-9}	4.43×10^{-7}	5.05×10^{-7}	2.86×10^{-6}
Romanesti	7.14×10^{-8}	1.71×10^{-8}	na	1.14×10^{-9}	2.71×10^{-7}	7.43×10^{-7}	2.86×10^{-6}
Stalpu	1.97×10^{-6}	1.29×10^{-8}	2.46×10^{-5}	1.43×10^{-9}	5.57×10^{-7}	6.86×10^{-7}	1.9×10^{-6}
Spataru	1.64×10^{-6}	1.43×10^{-8}	3×10^{-5}	2.86×10^{-9}	8×10^{-7}	8.29×10^{-7}	7.62×10^{-6}
Sinaia Lac	1.87×10^{-6}	1×10^{-8}	1.96×10^{-5}	2.29×10^{-9}	4.5×10^{-7}	5.71×10^{-7}	2.86×10^{-6}
Suseni Bilciuresti	3.36×10^{-6}	na	na	na	3.93×10^{-7}	6.1×10^{-7}	1.9×10^{-6}
Salcuta	2.57×10^{-6}	1.14×10^{-8}	1.79×10^{-5}	2×10^{-9}	3.43×10^{-7}	5.05×10^{-7}	1.9×10^{-6}
Sicrita	1.87×10^{-6}	4.29×10^{-9}	5.71×10^{-6}	1.71×10^{-9}	5.43×10^{-7}	6.38×10^{-7}	3.81×10^{-6}

* na = not applicable
